# Shared Transcriptomic Signatures and Network Interactions Between Lung Adenocarcinoma and Asthma

**DOI:** 10.3390/ijms27125544

**Published:** 2026-06-19

**Authors:** Seha Akduman, Elif Düz, Merve Gündoğdu, Didem Tecimel, Altay Burak Dalan, Ömer Faruk Bayrak, Didem Seven

**Affiliations:** 1 Department of Pulmonary Diseases, School of Medicine, Yeditepe University, Istanbul 34755, Türkiye; seha.akduman@yeditepe.edu.tr; 2 Department of Medical Genetics, School of Medicine, Yeditepe University, Istanbul 34755, Türkiye; elif.duz@yeditepe.edu.tr (E.D.); merve.gundogdu@std.yeditepe.edu.tr (M.G.); didem.tecimel@yeditepe.edu.tr (D.T.); ofbayrak@yeditepe.edu.tr (Ö.F.B.); 3 Department of Genetics and Bioengineering, Faculty of Engineering, Yeditepe University, Istanbul 34755, Türkiye; 4 Department of Biochemistry, School of Medicine, Yeditepe University, Istanbul 34755, Türkiye; abdalan70@yahoo.com

**Keywords:** asthma, lung adenocarcinoma (LUAD), co-expression network, protein interaction network (PPI), transcriptome

## Abstract

Lung adenocarcinoma (LUAD) remains the leading cause of mortality worldwide, while asthma is the most prevalent chronic disease affecting individuals of all ages. The shared airway involvement in these global health concerns results in exposure to common risk factors, suggesting a potential overlap in their genetic background. Given the roles oxidative stress and chronic inflammation play in both LUAD and asthma, we aimed to investigate similarities in their molecular mechanisms and to explore whether these shared transcriptomic signatures may prove useful for potential drug repurposing hypotheses by analyzing relevant transcriptomic datasets. This analysis identified a set of genes and co-expression interactions shared between asthma and LUAD, suggesting potential common molecular mechanisms underlying both diseases. Specifically, *DNAJC3*, *APOBEC3G*, and *PRDX4* were highlighted as potential common molecular mediators underlying both diseases, offering correlative evidence for shared pathways. These genes may represent key molecular links connecting the pathogenic processes of asthma and LUAD. The transcriptomic profiles of asthma and lung cancer datasets reveal common molecular interactions, suggesting potential shared biological mechanisms between the two diseases. These findings provide a computational framework that may guide future studies investigating therapeutic associations and possible drug–gene relationships in lung adenocarcinoma and asthma.

## 1. Introduction

Lung cancer remains the most frequent and lethal malignancy globally. Recent epidemiological data indicate that approximately 2.2 million new cases of lung cancer are diagnosed each year, constituting 11.4% of all cancer diagnoses worldwide. Furthermore, it accounts for an estimated 1.8 million deaths annually, representing 18% of all cancer-related mortalities on a global scale [1]. Though there have been significant advancements in diagnostic modalities and therapeutic interventions, the prognosis for lung cancer continues to be dismal. The overall 5-year survival rate is approximately 19%, with considerable variability depending on the stage at diagnosis, histological subtype, and access to healthcare resources. For patients with localized disease, the 5-year survival rate improves substantially to 56%. However, the prognosis for those with metastatic disease remains dire, with a 5-year survival rate of less than 5% [1]. These statistics highlight the critical need for enhanced strategies in primary prevention, early detection, and innovative treatment approaches. Comprehensive tobacco control policies, advancements in molecularly targeted therapies, and the development of novel immunotherapeutic agents are essential to improve outcomes for this devastating disease.

Asthma is a chronic respiratory disease marked by airway inflammation and hyper-responsiveness, affecting individuals globally and representing a major public health concern. Asthma affects approximately 262 million people worldwide, according to recent World Health Organisation (WHO) data, with rising prevalence. Rates are higher in developed regions compared to low-income areas. Annual incidence estimates vary but there are approximately 5–10 new cases per 1000 individuals in high-prevalence regions, emphasizing the need for standardized diagnostic criteria. Asthma is a leading cause of morbidity, with substantial healthcare and economic burdens. In 2019, asthma accounted for 461,000 global deaths, many of them preventable through appropriate management [2]. Recent evidence has demonstrated an association between asthma and an increased predisposition to several types of cancer, including breast, gastric, and lung malignancies [3,4]. The underlying mechanism is likely chronic inflammation, which plays a significant role in the pathogenesis of both conditions. Given the shared anatomical location in the upper airways, lung cancer holds particular importance in this association [5]. Risk factors contributing to this association can be categorized into two distinct groups: individual factors and environmental factors. Individual factors encompass tobacco exposure, genetic predisposition, obesity, and the presence of comorbidities. In contrast, environmental risk factors include air pollution, occupational hazards, climatic conditions, and allergen exposure. The association between lung cancer and asthma has long been a subject of debate among researchers. Notably, several studies have explored this relationship using diverse methodologies, including systematic literature reviews, patient data analyses, and genomic association studies [6,7,8].

While previous research has established epidemiological links between chronic airway inflammation (asthma) and lung adenocarcinoma (LUAD), the precise, shared genetic mechanisms and molecular intersections between these two conditions remain actively debated. Our study directly addresses this gap by utilizing an integrated transcriptomic approach to capture the conserved, shared Differentially Expressed Genes (DEGs) and molecular pathways that cross-talk between asthma and LUAD, establishing a concrete molecular framework for their clinical interplay.

Advancements in emerging transcriptomic technologies have enabled the identification of associations between distinct biological processes through large-scale gene expression profiling. These approaches provide an opportunity to explore shared molecular mechanisms underlying complex diseases. In this context, investigating the relationship between asthma and lung cancer may help to generate novel candidates regarding common biological pathways, particularly those related to chronic inflammation and oxidative stress. Drug repurposing (or drug repositioning) has emerged as a cost-effective and time-efficient strategy in drug discovery, especially in oncology; however, its application requires careful interpretation and strong experimental validation [9]. Although asthma medications are primarily developed to control airway inflammation, accumulating evidence suggests that some of these agents may influence biological processes that are also relevant to tumor development and progression. Chronic airway inflammation and LUAD share several signaling pathways, including NF-κB, PI3K/Akt, MAPK, JAK/STAT, and histamine receptor-mediated networks [10]. Consequently, asthma-related drugs may exert effects beyond inflammation control by modulating pathways involved in cell proliferation, apoptosis, immune regulation, oxidative stress responses, and therapeutic resistance. This biological overlap provides a rationale for exploring asthma-associated therapeutics within a drug-repurposing framework for LUAD.

In this study, we aimed to identify common transcriptomic signatures between asthma and LUAD using publicly available gene expression datasets. We further integrated asthma related drug–gene associations to explore potential molecular overlaps that may provide a framework for future studies investigating therapeutic relationships between the two diseases. Overall, this approach is intended to provide a systems-level, exploratory perspective on shared molecular mechanisms between asthma and LUAD rather than to propose definitive drug-repurposing candidates.

## 2. Results

**Figure 1:**
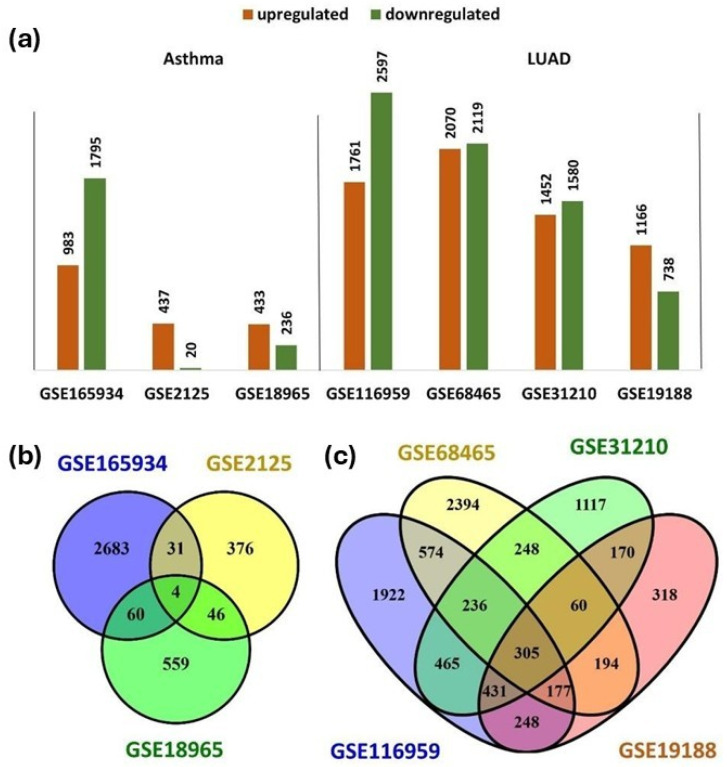
The number of differentially expressed genes for asthma and LUAD datasets. (**a**) shows the upregulated and downregulated genes and (**b**) represents the common DEGs between asthma datasets and (**c**) represents the common DEGs between LUAD datasets.

A large number of DEGs were identified in the LUAD datasets, whereas the number of DEGs in asthma datasets was relatively low (Figure 1a). In addition, asthma datasets exhibited lower internal consistency compared to LUAD datasets (Figure 1b). The variability among asthma datasets may be associated with differences in tissue sources, sample sizes, and probe platforms. Specifically, GSE165934 was derived from peripheral blood mononuclear cells (PBMCs), GSE2125 from alveolar macrophages, and GSE18965 from asthmatic epithelial tissue.

**Figure 2:**
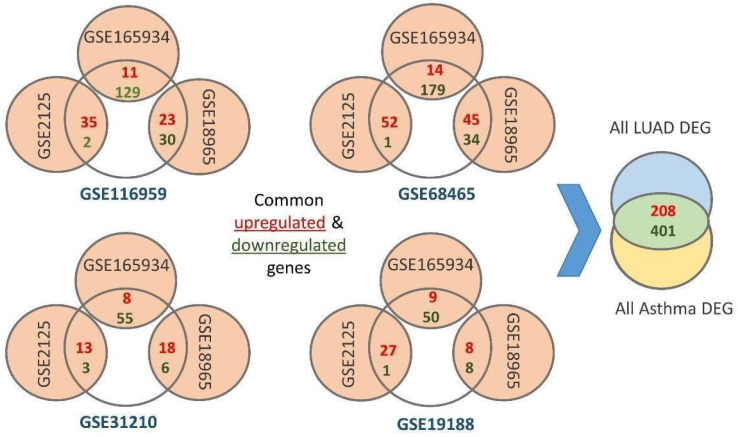
The common differentially expressed genes between LUAD and asthma datasets.

Due to the variability observed among asthma datasets, shared DEGs between asthma and LUAD datasets were analyzed individually. A total of 208 downregulated and 401 upregulated genes were identified as common between at least one LUAD and one asthma dataset (Figure 2; Online Resource S1).

**Figure 3:**
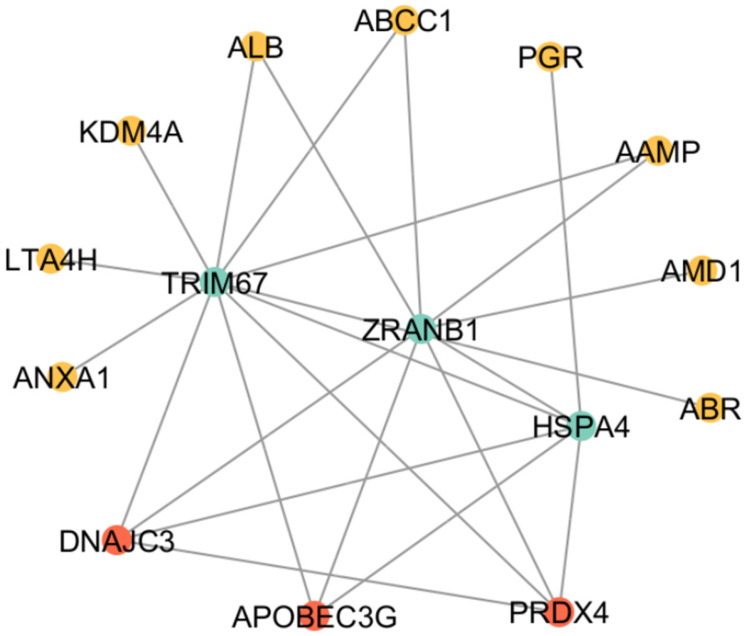
The PPI interaction of DEGs and drug-related genes. Green genes are downregulated, red genes are upregulated and yellow genes are drug-related genes.

The common genes identified across datasets and drug-related genes were integrated into a PPI analysis, resulting in 13,352 proteins and 92,147 interactions. Drug-related genes directly interacted with DEGs within the network (Figure 3). Among these genes, *NR3C1* and *POLK* were identified as both drug-related and downregulated genes.

**Figure 4:**
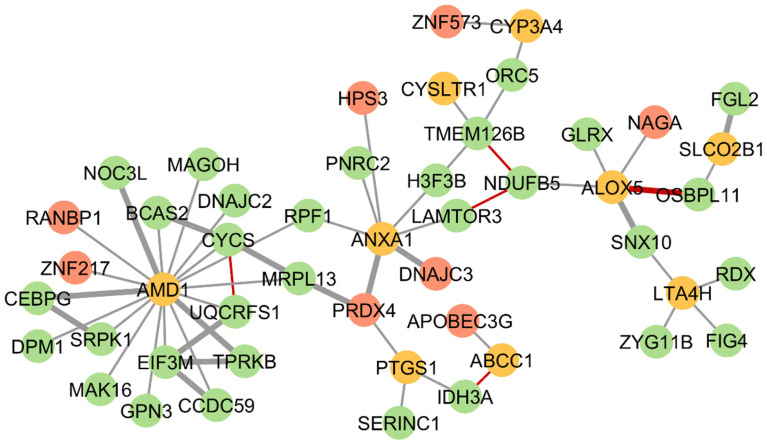
Co-expression interactions between drug-related genes and DEGs. Red edges represent interactions that are shared between the PPI and the co-expression network, black edges represent interactions derived exclusively from the co-expression network. The thickness of the edges indicates the frequency of each interaction across the datasets.

Co-expression network analysis of the common DEGs (*n* = 609) and drug-related genes (*n* = 57) identified 33 interactions shared across all three asthma datasets and 1616 interactions present in at least two asthma datasets. In LUAD datasets, 95 interactions were identified in at least two datasets. Among these, *CCDC59-HSPA13* and BCAS2-DDX21 interactions were consistently detected in both LUAD and asthma datasets (Online Resource S2). A total of 1277 interactions were identified in at least one LUAD and one asthma dataset, and 1193 of these interactions showed a consistent interaction pattern across datasets. Additionally, 63 interactions overlapped with the PPI (Figure 4; Online Resource S2).

**Figure 5:**
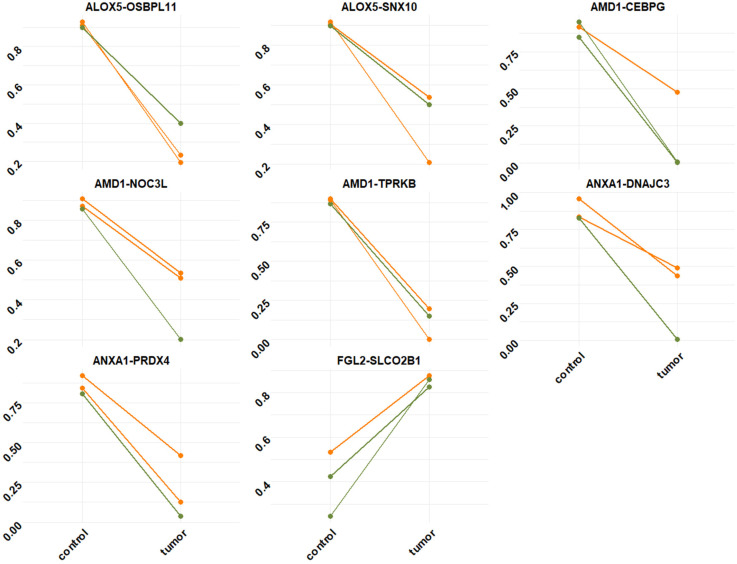
The correlation interactions for asthma and LUAD genes that were detected in more than two datasets. The orange lines belong to asthma datasets and green lines represent the LUAD datasets.

Several co-expression interactions involving drug-related genes and DEGs were consistently detected across multiple datasets and were also supported by the human PPI. The interaction between *ABCC1* and *IDH3A* was identified in both LUAD and asthma datasets and overlapped with the PPI. Similarly, *ALOX5* exhibited both PPI and co-expression interactions with *OSBPL11*, and these interactions were detected in multiple datasets (GSE2125, GSE18965, and GSE31210). *ANXA1* was found to interact with the upregulated genes *PRDX4* and *DNAJC3* in multiple datasets. In addition, *AMD1* showed consensus interactions with the downregulated genes *CEBPG* and *TPRKB* (Figure 5).

**Figure 6:**
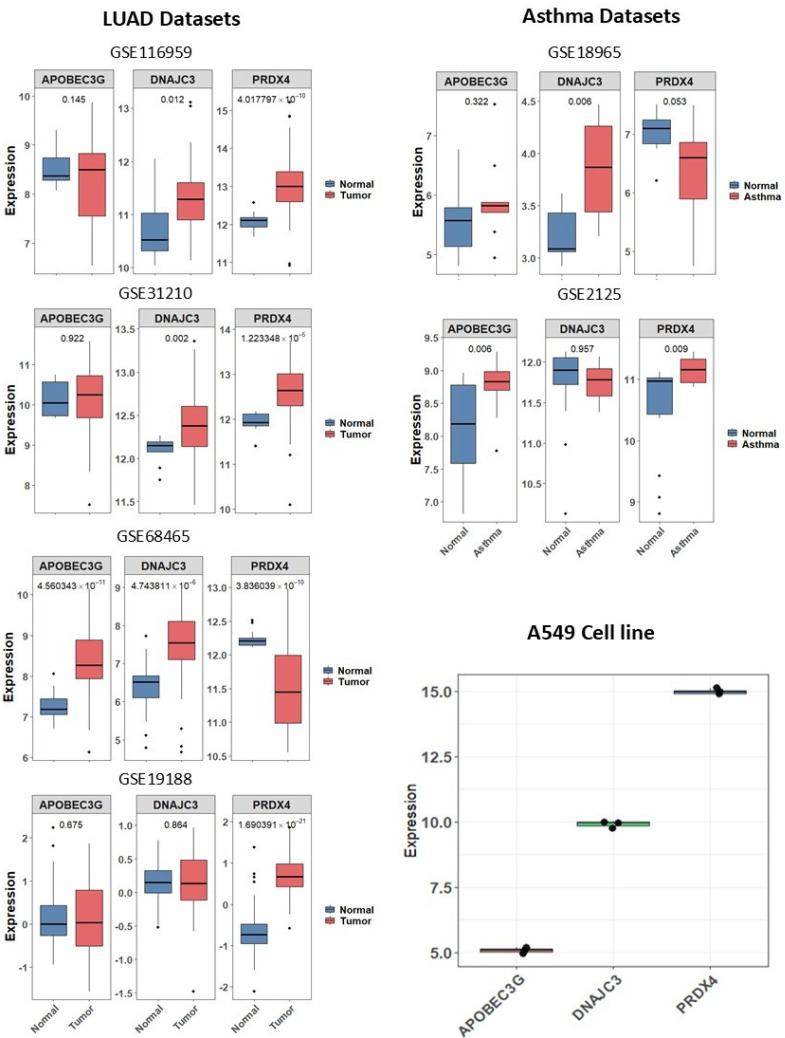
Expression profiles of selected genes across LUAD, asthma, and cell line datasets obtained from the GEO database.

Functional enrichment analysis of the commonly upregulated genes demonstrated enrichment in pathways associated with immune system processes, apoptosis, cytokine-mediated signaling, and oxidative stress (Online Resource S1). Several genes involved in these pathways, including *DNAJC3, APOBEC3G,* and *PRDX4,* also participated in the co-expression network and displayed upregulated expression patterns in both LUAD and asthma datasets (Figure 6).

**Figure 7:**
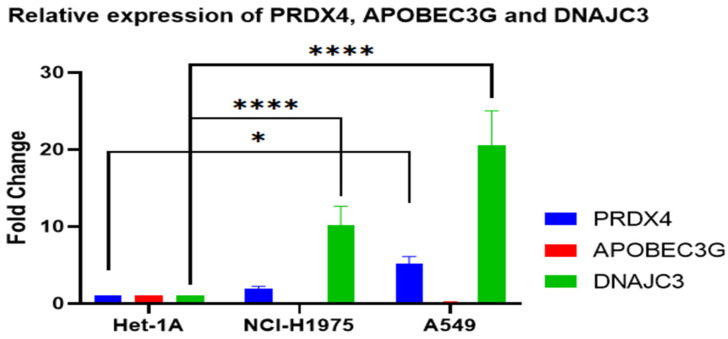
Relative expression of PRDX4, APOBEC3G, and DNAJC3 in lung cancer cell lines compared with normal Het-1A cells. (*: *p* < 0.05; ****: *p* < 0.0001).

Based on the transcriptomic analyses, qRT-PCR validation was performed in the LUAD cell lines A549 and NCI-H1975. *DNAJC3* and *PRDX4* showed significant upregulation in both lung cancer cell lines compared with the healthy epithelial cell line Het-1A. In contrast, *APOBEC3G* exhibited a downregulated expression pattern in both lung cancer cell lines (Figure 7). Overall, the experimental validation results confirmed the expression patterns of *DNAJC3* and *PRDX4* observed in the bioinformatic analyses, while *APOBEC3G* displayed an opposite expression pattern in vitro compared to transcriptomic datasets.

Univariate Cox regression analysis revealed that elevated APOBEC3G expression was significantly associated with poorer overall survival in the GSE19188 cohort (HR = 7.86, 95% CI: 1.66–37.14, *p* = 0.009). Consistently, Kaplan–Meier analysis demonstrated significantly reduced survival among patients with high APOBEC3G expression (log-rank *p* = 0.003). In contrast, *DNAJC3* and *PRDX4* showed no significant association with overall survival. In the independent GSE31210 cohort, none of the three genes reached statistical significance, although *DNAJC3* and *PRDX4* exhibited trends toward increased risk. Notably, *APOBEC3G* displayed an opposite effect direction in GSE31210 (HR = 0.33), indicating the limited reproducibility of its prognostic impact across datasets (Table 1).

## 3. Discussion

This study demonstrates shared transcriptional and interactional signatures between asthma and LUAD, highlighting common molecular alterations across inflammatory and malignant lung conditions. Figure 1 suggests that detecting asthma based on expression data may be more challenging (Figure 1a). Additionally, asthma datasets exhibit low internal consistency, which may be attributed to variations in tissue sources, limited sample sizes, or differences in the probes used (Figure 1b). In contrast, the comparison between tumor and healthy tissues in LUAD datasets yielded a much higher number of significant DEGs compared to the asthma datasets. Moreover, because LUAD datasets are generally derived from similar tissue types, the identified DEGs show a high degree of consistency across datasets. Moreover, the larger sample size in LUAD datasets, compared to asthma datasets, contributes to these differences in DEG identification. However, the asthma datasets contain less common genes due to variability in sampling methods. For instance, GSE165934 is derived from peripheral blood mononuclear cells (PBMCs) of asthma patients, GSE2125 is obtained from alveolar macrophages, and GSE18965 profiles asthmatic epithelial tissue (Figure 1).

Given the variability within asthma datasets, we focused on the shared DEGs between asthma and LUAD. Since asthma datasets did not exhibit strong internal concordance, we compared each asthma dataset individually with the DEGs identified in each LUAD dataset. Through this comparison, a total of 208 downregulated and 401 upregulated genes were found to be shared between at least one LUAD and one asthma dataset (Figure 2) (Online Resource S1). Supporting this evidence, a study investigating the association between atopy, asthma, and cancer in a large cancer cohort suggested a potential interaction between lung cancer and asthma [11].

The common genes identified across datasets were combined with drug-related genes, and a PPI was constructed using these genes along with their direct interaction partners. This network comprised 13,352 proteins and 92,147 interactions. Drug-related genes were directly interacted with DEGs (Figure 3). *NR3C1* and *POLK* are both drug-related and downregulated genes. The *NR3C1* gene encodes glucocorticoid receptors and polymorphisms in the *NR3C1* gene may differentially influence TGF-β mRNA expression and contribute to the fibrotic process in the lungs of asthmatic patients [12]. *NR3C1* downregulation was observed in LUAD patients and negatively correlated with overall survival [13]. Additionally, this is the first study to highlight the downregulation of the *POLK* gene. The encoded protein is a specialized DNA polymerase that facilitates translation DNA synthesis, allowing DNA replication to proceed despite the presence of DNA lesions [14]. Notably, the absence of this gene has been reported to contribute to oxidative damage [15]. Given the well-established link between oxidative stress and inflammation in lung cancer and asthma, the downregulation of *POLK* may play a critical role in disease progression [16].

Common differentially expressed genes (*n* = 609) and drug-related genes (*n* = 57) across all datasets were integrated, and a co-expression network analysis was performed. A total of 33 interactions were shared across all three asthma datasets, while 1,616 interactions were present in at least two asthma datasets. In the LUAD datasets, no interactions were found to be common across all four datasets; however, 95 interactions were identified in at least two LUAD datasets. Notably, two interactions, CCDC59-HSPA13 (observed as a negative correlation in three datasets and a positive correlation in one dataset) and BCAS2-DDX21 (negative correlation in all datasets), were consistently observed in at least two LUAD and two asthma datasets, with BCAS2-DDX21 exhibiting the same interaction pattern across conditions (Online Resource S2). A detailed analysis of all LUAD and asthma interactions identified 1,277 interactions present in at least one LUAD and one asthma dataset. Among these, 1193 interactions exhibited a consistent pattern across datasets (either increasing or decreasing) and 63 of them overlapped with PPI (Figure 4) (Online Resource S2). Previous studies have demonstrated that the TTF1-CCDC59 complex plays a crucial role in lung tissue by modulating alveolar surface tension, which is essential for maintaining proper lung function and gas exchange [17]. *CCDC59* gene that functions in RNA binding activity was previously reported as a lung tumor biomarker [18]. However, there is limited information about that gene. *HSPA13*, one of the 13 members of HSP70 with various immunomodulatory properties and distinct activities in airway inflammatory processes and asthma, interacts with *CCD59* [19]. *BCAS2* gene (Breast cancer amplified sequence 2) plays a key role in mRNA splicing and overexpression of the gene was reported in breast cancer [20]. BCAS2 was observed to interact with *DDX21*, a DEAD-box RNA helicase involved in modulating RNA secondary structure. *DDX21* plays a key role in fundamental cellular processes, including translation initiation, nuclear and mitochondrial splicing, as well as ribosome and spliceosome assembly [21]. A recent pan-cancer study identified *DDX21* gene amplification in LUAD samples, which was associated with poor prognosis [22]. In contrast, our findings suggest that the decreased interaction between these two genes is linked to LUAD and asthma. This may be due to their interaction influencing distinct molecular mechanisms compared to DDX21 alone.

Co-expression network analysis reveals interactions between drug-related genes and DEGs, with some interactions consistently observed in more than two datasets. Additionally, several of these co-expression interactions correspond to known protein–protein interactions (Figure 4). *ABCC1*, a gene associated with Montelukast Sodium, interacts with IDH3A, which is downregulated in both LUAD and asthma datasets. This interaction is also present in the human PPI. The *ABCC1* gene that encodes multidrug resistance associated with protein 1 (MRP1), a member of ABC subfamily C1, was first identified in small-cell lung cancer cell line H69AR [23,24]. *ABCC1* overexpression occurs in various hematological and solid tumors. Tumors from patients with conditions such as non-small-cell lung carcinoma (NSCLC) or chronic lymphocytic leukemia (CLL) generally exhibit high *ABCC1* expression levels and resistance to multiple chemotherapeutic agents. In contrast, *ABCC1* overexpression is reported to be less common in small-cell lung carcinoma (SCLC), colorectal carcinoma, neuroblastoma, and retinoblastoma [25,26,27]. On the other hand, overexpression of *IDH3α* confers resistance to chemoimmunotherapy [28]. Previously, it was reported that high IDH3A expression is associated with reduced postoperative overall survival in lung and breast cancer patients [29]. Very recently it was reported that ABCC1 mRNA, and protein levels were found to be elevated in human airway smooth muscle cells [30]. Yeung et al. investigated isocitrate dehydrogenases in asthmatic and non-asthmatic individuals and found no significant difference in *IDH3A* gene expression [31]. Our study demonstrates that the interaction between *ABCC1* and *IDH3A* is associated with both asthma and lung cancer, suggesting a potential molecular link between these conditions.

*ALOX5*, another gene related to Montelukast Sodium, exhibits both PPI and co-expression interactions with *OSBPL11*, a downregulated gene. This interaction was detected in more than two datasets (GSE2125, GSE18965, GSE31210). ALOX5 mRNA level downregulated in adenocarcinoma and squamous cell carcinoma of the lung, and this was positively correlated with immune infiltration of tumor cells [32]. The polymorphism in the promoter region of this gene (*ALOX5* or *OSBPL11*) was found to be associated with the response to montelukast in patients with persistent atopic asthma [33]. Later, it was also demonstrated that leukotriene production reduces asthma in children [34]. The *OSBPL11* gene mutation has previously been associated with lung cancer. Our study is the first to report the downregulation of *OSBPL11* in lung cancer and its interaction with *ALOX5* [35].

*ANXA1*, associated with Budesonide, interacts with two upregulated genes, *PRDX4* and *DNAJC3*. These interactions were observed in multiple datasets (ANXA1-PRDX4: GSE2125, GSE18965, GSE31210; ANXA1-DNAJC3: GSE165934, GSE2125, GSE31210). *ANXA1* is an anti-inflammatory protein that plays a crucial role in protecting cells from oxidative stress in the respiratory system [36]. It has been associated with the asthma medication budesonide, a glucocorticoid that regulates the expression of anti-inflammatory pathways [37]. Our findings demonstrated that *ANXA1* interacts with the *PRDX4* and *DNAJC3* genes. *PRDX4* plays a vital role in managing oxidative stress by decreasing the hydrogen peroxide level in the endoplasmic reticulum [38]. The overexpression of *PRDX4* has been reported in several cancers, including gastric, pancreatic, prostate, lung, colorectal, ovarian and oral cavity cancers [39,40]. *AMD1* shows consensus interactions with two downregulated genes, *CEBPG* and *TPRKB.* The AMD1-CEBPG interaction was identified in the GSE2125, GSE116959, and GSE68465 datasets, while the AMD1-TPRKB interaction was found in GSE2125, GSE18965, and GSE116959 (Figure 5).

The functional enrichment analysis was also performed for the commonly upregulated genes. These genes are involved in asthma- and lung cancer-related pathways, including immune system processes, apoptosis, cytokine-mediated signaling, oxidative stress, and others (Online Resource S1). Genes associated with these pathways were examined in detail, and several such as DNAJC3, APOBEC3G, and PRDX4 also showed interactions within the correlation network. Notably, these genes exhibit an upregulated expression pattern in both LUAD and asthma samples (Figure 6). Since the asthma datasets originate from different tissue sources, and at least one dataset showed that these genes are upregulated compared to control samples, we examined the expression levels of these genes in a LUAD cell line (A549) (Figure 5). This analysis aimed to confirm that these genes are expressed in cell lines as well, indicating their applicability for experimental validation.

Some of these genes show significant changes based on survival analysis. *DNAJC3* gene is upregulated in at least one lung and one asthma datasets and high expression of the *DNAJC3* gene is associated with a lower survival rate in lung cancer (Online Resource S4). The upregulation of this gene has been observed in breast cancer, and its expression has been reported to be regulated by miRNA-144 [41]. *APOBEC3G* is one of the upregulated genes, and its higher expression levels are associated with a lower survival rate in lung cancer (Online Resource S4). Previous studies have demonstrated an association between *APOBEC3G* gene mutations and lung cancer [42]. In our dataset, high expression of *APOBEC3G* was observed in both datasets. *PRDX4* is also one of the common upregulated genes and its upregulation is related to the lower survival rate (Online Resource S4). Upregulation of this gene has been associated with increased proliferation of lung cancer cells in the A549 cell line [43]. *APOBEC3G* is also one of the common upregulated genes, and its knockdown in A549 cells enhances radiation-induced DNA damage, suggesting that its inhibition may improve radiotherapy efficacy in lung cancer [44].

Based on the transcriptomic analyses of GEO datasets, quantitative real-time PCR (qRT-PCR) was performed to experimentally validate the expression patterns of selected candidate genes in lung cancer cell lines. Consistent with the bioinformatics findings, *DNAJC3* and *PRDX4* exhibited significant upregulation in the lung cancer cell lines A549 and NCI-H1975 compared with the healthy human epithelial cell line Het-1A. In contrast, *APOBEC3G* showed a downregulated expression pattern in both lung cancer cell lines (Figure 7). The concordant expression of *DNAJC3* and *PRDX4* between in silico and in vitro analyses supports the robustness of the bioinformatic approach and suggests that these genes may represent biologically relevant molecular features shared between asthma and LUAD. In particular, *PRDX4* upregulation is in line with previous studies reporting its increased expression in LUAD and its functional association with EGFR mutation status. Consistent with our findings showing *PRDX4* upregulation in lung cancer cell lines, Mizutani et al. reported increased *PRDX4* expression in LUAD models and further demonstrated that its biological effects are closely linked to EGFR mutation status. *PRDX4* overexpression suppressed cellular proliferation in EGFR wild-type A549 cells, whereas this growth-inhibitory effect was markedly reduced in EGFR-mutant cell models. Notably, *PRDX4* upregulation in EGFR-mutant PC-9 cells did not result in significant changes in proliferative capacity, suggesting that constitutively active EGFR signaling may override redox-mediated growth regulation. Collectively, these findings indicate that the biological function of *PRDX4* in LUAD is highly dependent on EGFR mutation status [45]. Moreover, Hao, Y. et al. demonstrated in transgenic mouse models that activation of the Prx4–Srx axis promotes lung adenocarcinoma development by disrupting redox homeostasis and shaping a pro-tumorigenic microenvironment. Consistently, these findings support a role for *PRDX4* as a context-dependent oncogenic factor in LUAD and highlight this pathway as a potential therapeutic and biomarker target [46]. Although *APOBEC3G* was identified as an upregulated gene in both our bioinformatic analyses and previous transcriptomic studies of lung cancer [47], qRT-PCR analysis revealed a downregulated expression pattern in lung cancer cell lines. This apparent discrepancy likely reflects the cellular composition of bulk tissue samples. *APOBEC3G* is highly expressed in tumor-infiltrating lymphocytes, macrophages, and other immune cells. Therefore, the elevated expression observed in tissue derived datasets may largely originate from the immune microenvironment rather than from the cancer cells themselves. In contrast, cell line models lack this immune component, thereby revealing the intrinsically lower expression of *APOBEC3G* in LUAD cells. This divergence may indicate context dependent regulation of *APOBEC3G*, potentially influenced by differences between bulk tissue-based datasets and in vitro cell line models. Consistent with this interpretation, members of the APOBEC family are known to play important roles in immune responses as well as cancer-associated mutational processes [48]. Our findings suggest that APOBEC3G expression may be dynamically regulated during lung tumorigenesis and may require further investigation to clarify its functional relevance in LUAD. To the best of our knowledge, no studies to date have directly investigated the role of *DNAJC3* in cancer or inflammatory respiratory diseases. In contrast, multiple studies have focused on DNAJC3-AS1, an antisense long non-coding RNA transcribed from the same genomic locus. Ni et al. demonstrated that increased DNAJC3-AS1 expression is associated with enhanced cell proliferation and a malignant phenotype in papillary thyroid carcinoma [49]. Similarly, Li et al. reported that high DNAJC3-AS1 expression promotes cell proliferation in acute myeloid leukemia [50]. In breast cancer, elevated DNAJC3-AS1 levels were linked to advanced disease stage, lymph node metastasis, and poor survival, and silencing of DNAJC3-AS1 suppressed cell proliferation, invasion, and epithelial–mesenchymal transition [51]. Although antisense long non-coding RNAs are often reported to negatively regulate their sense transcripts, this relationship is not universal and can be highly context-dependent [52]. In the present study, *DNAJC3* expression was found to be upregulated in tumor tissues, suggesting a potential co-regulation of *DNAJC3* and DNAJC3-AS1 under tumor-associated stress conditions. Given the established role of *DNAJC3* in endoplasmic reticulum stress and unfolded protein response pathways, its increased expression may reflect an adaptive mechanism that supports tumor cell survival. Therefore, concurrent upregulation of DNAJC3 and DNAJC3-AS1 cannot be excluded and may indicate complementary, rather than antagonistic, roles in cancer biology. Future studies are needed to clarify the functional relationship between DNAJC3 and DNAJC3-AS1 and to better understand their roles in cancer biology. Collectively, *DNAJC3*, *APOBEC3G*, and *PRDX4* are involved in cellular stress adaptation, encompassing endoplasmic reticulum stress, oxidative stress regulation, and genomic stability. The altered expression of these genes in lung cancer cells supports the hypothesis that chronic stress-related pathways constitute a shared molecular framework between asthma and LUAD.

These findings highlight potential gene interactions and drug-related genes that are consistently observed across currently available datasets, suggesting their relevance in both LUAD and asthma. Thus, our study paves the way for identifying currently approved molecules used for other conditions, such as asthma or allergies, that can be repurposed for anticancer therapy. Functional assays assessing the anticancer properties of asthma drugs were not conducted in the present study. Our analyses are limited to transcriptomic correlations, co-expression networks, and qRT-PCR validation of gene expression levels. While we identify candidate genes (e.g., *ABCC1, ALOX5, ANXA1*) and interactions that could be targeted by asthma-related drugs (e.g., montelukast, budesonide), we have not performed any direct drug treatment experiments on LUAD cell lines [53,54]. Therefore, our findings should be considered as hypothesis-generating rather than evidence of therapeutic efficacy. Future studies are required to test whether these drugs affect LUAD cell proliferation, migration, invasion, or apoptosis using functional assays such as MTT, wound healing, or transwell assays. The present study provides a molecular rationale for such experiments but does not replace them. Beyond this, the rationale for exploring asthma-related therapeutics in LUAD extends beyond the suppression of peritumoral inflammation alone. Emerging evidence suggests that chronic airway inflammatory disorders and lung cancer may share overlapping molecular networks involved in immune regulation, oxidative stress responses, cell survival, and tissue remodeling. Consequently, certain asthma- and allergy-related medications may influence cancer-relevant processes such as cell proliferation, apoptosis, and cancer stemness by modulating these shared pathways [55]. Indeed, previous studies have reported that agents such as budesonide, ciclesonide, and antihistamines can modulate tumor suppressor pathways, stemness-associated signaling networks, and antitumor immune responses [53,56]. Therefore, the potential repurposing value of these drugs may arise not only from their anti-inflammatory properties but also from their ability to target molecular mechanisms involved in malignant progression. Nevertheless, whether these effects translate into clinically meaningful antitumor activity in LUAD remains to be determined through dedicated functional and mechanistic studies.

A limitation of this study is the heterogeneous tissue origin of the analyzed datasets. While the LUAD datasets were derived from tumor tissues, the asthma datasets included samples obtained from different biological sources, including peripheral blood mononuclear cells, alveolar macrophages, and airway epithelial tissues. Consequently, some of the observed transcriptomic alterations may reflect tissue-specific expression patterns, cellular composition differences, or microenvironmental effects rather than disease-specific mechanisms alone. To minimize potential batch effects and tissue-related biases, datasets were analyzed independently rather than combined into a single expression matrix, and only reproducible signatures shared across datasets were considered in downstream analyses. We reasoned that molecular alterations consistently detected across distinct biological contexts may represent robust disease-associated mechanisms. Nevertheless, the identified shared genes and pathways should be interpreted with caution, as the contribution of tissue-specific factors cannot be completely excluded. Therefore, further validation using matched tissue types, larger cohorts, and single-cell or spatial transcriptomic approaches will be necessary to confirm the biological relevance of the proposed shared biomarkers and mechanisms between asthma and LUAD.

To ensure our study moves beyond descriptive, simple co-expression, we established a multi-step pipeline that transitions from in silico discovery to clinical trend analysis and in vitro experimental verification. Rather than just listing correlated genes, we merged the shared up- and downregulated DEGs directly with verified asthma-related drug target genes. Through protein–protein interaction and correlation networks, we demonstrated that these genes are directly or indirectly interconnected, maintaining a conserved regulatory pattern in at least one asthma and one LUAD dataset. To explore the clinical relevance of these interconnected network components, we evaluated their behavior in independent LUAD survival datasets. Although these candidate genes did not reach strict statistical significance (*p* < 0.05) in the survival models, they exhibited a consistent, noteworthy prognostic pattern where higher expression levels clinically trended toward poorer overall survival. We evaluated this directional pattern as a hypothesis-generating signal rather than a definitive statistical association. Also, to ground these computational predictions and survival trends in a biological system, we selected three key genes exhibiting this consistent pattern and validated their expression profiles in LUAD cell cultures using qPCR.

In summary, our study goes beyond just showing that genes change together. By combining data analysis, drug-target networks, survival trends, and laboratory experiments, we did not just describe a correlation. Instead, we used computational findings as a starting point to discover and experimentally verify the actual molecular links between asthma and lung adenocarcinoma.

## 4. Materials and Methods

In this study, we performed differentially expressed gene analysis for asthma and LUAD datasets separately and identified the common deregulated genes between them. The workflow of the study is shown in the graphical abstract.

Differential gene expression analysis was conducted separately for each dataset using the limma package, with significantly differentially expressed genes identified based on an adjusted *p*-value threshold of <0.05 and a fold change threshold of >1.5. For downstream cross-dataset analysis, differential expression results were first generated independently for each LUAD and asthma datasets, and then used in pairwise comparisons between each LUAD dataset and each asthma dataset to identify overlapping DEGs. Shared gene sets derived from all pairwise comparisons were subsequently aggregated to obtain a final list of reproducible LUAD–asthma common DEGs. No batch-effect correction was applied, as datasets were analyzed independently without integration. Instead, robustness was addressed by focusing on genes that were consistently identified across multiple independent LUAD–asthma comparisons.

### 4.1. Data Description and Preprocessing

Three asthma and four LUAD datasets were obtained from the GEO database. The datasets were already normalized; for un-normalized data, log2 transformation was applied. The characteristics of the datasets are summarized in Table 2. In total, 35 control and 31 asthma samples, as well as 102 control and 233 LUAD samples, were analyzed.

Each dataset underwent a preprocessing pipeline, where Probe IDs were mapped to gene symbols, and for genes with identical symbols, the maximum expression value was retained. Outlier detection was performed using principle component analysis (PCA) with prcomp function in R, leading to the removal of three tumor samples (GSM475677, GSM475780, GSM475706) and one control sample (GSM475666) from the GSE19188 dataset. Differential gene expression analysis was conducted using the limma package, with significantly differentially expressed genes identified based on an adjusted *p*-value threshold of <0.05 and a FC threshold of >1.5. For validation, the GSE117036 dataset (derived from A549 LUAD cells) was used to assess the expression levels of the selected genes. All analyses were performed using the R programming language (version 4.4.0).

Related gene lists for six different asthma drugs (Budesonide, Fluticasone, Umeclidinium Bromide, Montelukast Sodium and Vilanterol) were obtained using the DrugBank and Drug Gene Interaction Database (DGIdb) [57,58].

### 4.2. Co-Expression Network Analysis

Common differentially expressed genes between LUAD and asthma datasets were identified and merged with drug-related genes. Correlation values for these genes in control and disease conditions were calculated using the cor function in R. Thresholds were defined for significantly increasing and decreasing interactions, with a minimum value of the maximum correlation value was set to 0.8 and a minimum correlation difference between control and disease samples was defined as 0.3. Based on these criteria, gene pairs exhibiting a shared pattern in LUAD and asthma datasets were identified. This analysis was performed separately for each of the seven datasets, and the results were aggregated and scored based on the frequency of interaction occurrence across datasets.

### 4.3. PPI Analysis

The human PPI was downloaded from the BIOGRID database (version 4.4.240), including only human-specific and physical interactions [59]. The direct interactions between DEGs and drug target genes were identified using the PPI.

### 4.4. Survival Analysis

Survival analysis was performed for DEGs using survival data from LUAD datasets that include survival information (GSE31210, GSE19188). The analysis was conducted using the survfit function in R, and a significance threshold of 0.05 was applied. For three selected genes, the hazard ratios (HRs), 95% confidence intervals (CIs), and corresponding *p*-values obtained from the Cox proportional hazards regression analyses were visualized using forest plots. Forest plots were generated to illustrate the prognostic effects and confidence intervals of the analyzed variables. All visualizations were created in R software (ver. 4.4.1) using the forestplot, survminer, and ggplot2 package.

### 4.5. Experimental Validation

#### 4.5.1. RNA Extraction and cDNA Synthesis

A549 and NCI-H1975 lung cancer cell lines and the healthy control cell line Het-1A were maintained in complete high-glucose DMEM (Gibco, Waltham, MA, USA) supplemented with 10% fetal bovine serum and 1% penicillin–streptomycin at 37 °C containing 5% CO_2_. Total RNA was extracted from cell pellets using TRIzol reagent (Invitrogen, Life Technologies, Carlsbad, CA, USA) in accordance with the manufacturer’s guidelines. RNA concentration and purity were measured with a NanoDrop ND-1000 spectrophotometer (Thermo Fisher Scientific, Waltham, MA, USA). For cDNA synthesis, 1000 ng of total RNA was reverse-transcribed in a 20 µL reaction volume using the High-Capacity cDNA Reverse Transcription Kit (Applied Biosystems, Thermo Fisher Scientific, Waltham, MA, USA), together with random primers and an RNase inhibitor, following the supplier’s protocol.

#### 4.5.2. qRT-PCR

qRT-PCR was carried out using a LightCycler^®^ 480 Instrument II (Roche, Basel, Switzerland). Sybr-Green based gene expression assays were used for the detection of target genes, and all reactions were performed in triplicate. An equal amount of cDNA (15 ng) was used in each reaction. PCR amplification and real-time fluorescence detection were conducted under the following thermal cycling conditions: an initial denaturation at 95 °C for 10 min, followed by 40 cycles of 95 °C for 15 s and 59 °C for 1 min. β-actin was used as the endogenous reference gene for normalization. All experiments were independently repeated at least three times. All primer sequences used in this study are provided in Table 3.

## Figures and Tables

**Table 1:** Univariate Cox proportional hazards and log-rank analysis of candidate genes across independent LUAD datasets.

Dataset ID	Gene	Hazard Ratio (HR)	95% CI (Low–High)	Cox *p*-Value	Log-Rank *p*-Value
GSE19188	DNAJC3	2.548642	0.77–8.49	0.128	0.113
	APOBEC3G	7.863697	1.66–37.14	0.009	0.003
	PRDX4	0.941411	0.30–2.93	0.917	0.926
GSE31210	DNAJC3	2.965016	0.60–14.70	0.183	0.162
	APOBEC3G	0.328455	0.07–1.63	0.174	0.152
	PRDX4	3.431	0.69–17.01	0.131	0.108

**Table 2:** The characteristics of LUAD and asthma datasets.

DatasetID	Sample Size	Probe	Normalization
GSE165934	9 control/11 asthma	Affymetrix Human Clariom D Assay	log2
GSE2125	15 control/15 asthma	Affymetrix Human Genome U133 Plus 2.0 Array	RMA normalized
GSE18965	7 control/9 asthma	Affymetrix Human Genome U133A Array	RMA normalized
GSE116959	11 control/57 LUAD	Agilent-039494 SurePrint G3 Human GE v2 8 × 60K Microarray 039381	log2
GSE68465	19 control/50 LUAD	Affymetrix Human Genome U133A Array	log2
GSE31210	8 control/84 LUAD	Affymetrix Human Genome U133 Plus 2.0 Array	log2
GSE19188	64 control/42 LUAD	Affymetrix Human Genome U133 Plus 2.0 Array	RMA normalized
GSE117036	3 A549 Cell line	Agilent-039494 SurePrint G3 Human GE v2 8 × 60K Microarray 039381	log2

**Table 3:** Primer sequences used for qPCR analysis.

Primer Name	Sequence 5′-3′
ACTB_F	GATCAAGATCATTGCTCCTCCT
ACTB_R	CTGATCCACATCTGCTGGAA
PRDX4_F	GGTTCAAGCATTCCAGTACACT
PRDX4_R	GGATCTGGGATTATTGTTTCACTA
APOBEC3G_F	GTTATGAGGTGGAGCGCAT
APOBEC3G_R	GGAGCCTGGTTGCATAGAA
DNAJC3_F	CTTCACTGCAGCAAGATTACAGA
DNAJC3_R	ACTTGGATTAGATTTGAGCACTT

## Data Availability

The data presented in this study can be downloaded from NCBI GEO database. NCBI. GEO [online]. Website: https://www.ncbi.nlm.nih.gov/geo/ [accessed 31 January 2025]. PPI interaction network was downloaded from BioGRID database. BioGRID [online]. Website: https://thebiogrid.org/ [accessed 31 January 2025].

## Supplementary Materials

The following supporting information can be downloaded at: https://www.mdpi.com/article/10.3390/ijms27125544/s1.

## References

[1] Sung H., Ferlay J., Siegel R.L., Laversanne M., Soerjomataram I., Jemal A., Bray F. (2021). Global Cancer Statistics 2020: GLOBOCAN Estimates of Incidence and Mortality Worldwide for 36 Cancers in 185 Countries. CA Cancer J. Clin..

[2] Wang Z., Li Y., Gao Y., Fu Y., Lin J., Lei X., Zheng J., Jiang M. (2023). Global, regional, and national burden of asthma and its attributable risk factors from 1990 to 2019: A systematic analysis for the Global Burden of Disease Study 2019. Respir. Res..

[3] Merrill R.M., Isakson R.T., Beck R.E. (2007). The association between allergies and cancer: What is currently known?. Ann. Allergy Asthma Immunol..

[4] Woo A., Lee S.W., Koh H.Y., Kim M.A., Han M.Y., Yon D.K. (2021). Incidence of cancer after asthma development: 2 independent population-based cohort studies. J. Allergy Clin. Immunol..

[5] Jiang L., Sun Y.-Q., Langhammer A., Brumpton B.M., Chen Y., Nilsen T.I., Leivseth L., Wahl S.G.F., Mai X.-M. (2021). Asthma and asthma symptom control in relation to incidence of lung cancer in the HUNT study. Sci. Rep..

[6] Qu Y.-L., Liu J., Zhang L.-X., Wu C.-M., Chu A.-J., Wen B.-L., Ma C., Yan X., Zhang X., Wang D.-M. (2017). Asthma and the risk of lung cancer: A meta-analysis. Oncotarget.

[7] Rosenberger A., Bickeböller H., McCormack V., Brenner D.R., Duell E.J., Tjønneland A., Friis S., Muscat J.E., Yang P., Wichmann H.-E. (2012). Asthma and lung cancer risk: A systematic investigation by the international lung cancer consortium. Carcinogenesis.

[8] Santillan A.A., Camargo C.A., Colditz G.A. (2003). A meta-analysis of asthma and risk of lung cancer (United States). Cancer Causes Control.

[9] Pushpakom S., Iorio F., Eyers P.A., Escott K.J., Hopper S., Wells A., Doig A., Guilliams T., Latimer J., McNamee C. (2018). Drug repurposing: Progress, challenges and recommendations. Nat. Rev. Drug Discov..

[10] Mishra V., Banga J., Silveyra P. (2018). Oxidative stress and cellular pathways of asthma and inflammation: Therapeutic strategies and pharmacological targets. Pharmacol. Ther..

[11] Kantor E.D., Hsu M., Du M., Signorello L.B. (2019). Allergies and asthma in relation to cancer risk. Cancer Epidemiol. Biomark. Prev..

[12] Panek M., Pietras T., Fabijan A., Zioło J., Wieteska Ł., Małachowska B., Fendler W., Szemraj J., Kuna P. (2015). The NR3C1 Glucocorticoid Receptor Gene Polymorphisms May Modulate the TGF-beta mRNA Expression in Asthma Patients. Inflammation.

[13] Chiang H.-H., Ong C.-T., Chang C.-Y., Wu K.-L., Wu Y.-Y., Lai J.-C., Shen T.-Y., Hung J.-Y., Lee H.-C., Tsai Y.-M. (2024). Downregulated antisense lncRNA ENTPD3-AS1 contributes to the development of lung adenocarcinoma. Am. J. Cancer Res..

[14] Xiang Y., Laurent B., Hsu C.-H., Nachtergaele S., Lu Z., Sheng W., Xu C., Chen H., Ouyang J., Wang S. (2017). RNA m6A methylation regulates the ultraviolet-induced DNA damage response. Nature.

[15] Michiels S., Danoy P., Dessen P., Bera A., Boulet T., Bouchardy C., Lathrop M., Sarasin A., Benhamou S. (2007). Polymorphism discovery in 62 DNA repair genes and haplotype associations with risks for lung and head and neck cancers. Carcinogenesis.

[16] Klaunig J.E. (2018). Oxidative stress and cancer. Curr. Pharm. Des..

[17] Islam A.B.M.M.K., Khan M.A.-A.-K. (2020). Lung transcriptome of a COVID-19 patient and systems biology predictions suggest impaired surfactant production which may be druggable by surfactant therapy. Sci. Rep..

[18] Andus I., Prall F., Linnebacher M., Linnebacher C.S. (2023). Establishment, characterization, and drug screening of low-passage patient individual non-small cell lung cancer in vitro models including the rare pleomorphic subentity. Front. Oncol..

[19] Shevchenko M., Servuli E., Albakova Z., Kanevskiy L., Sapozhnikov A. (2021). The Role of Heat Shock Protein 70 kDa in Asthma. J. Asthma Allergy.

[20] Maass N., Rösel F., Schem C., Hitomi J., Jonat W., Nagasaki K. (2002). Amplification of the BCAS2 gene at chromosome 1p13. 3–21 in human primary breast cancer. Cancer Lett..

[21] Nagasaki K., Maass N., Manabe T., Hanzawa H., Tsukada T., Kikuchi K., Yamaguchi K. (1999). Identification of a novel gene, DAM1, amplified at chromosome 1p13. 3-21 region in human breast cancer cell lines. Cancer Lett..

[22] Hu A., Wang Y., Tian J., Chen Z., Chen R., Han X., Chen Y., Liu T., Chen Q. (2022). Pan-cancer analysis reveals DDX21 as a potential biomarker for the prognosis of multiple tumor types. Front. Oncol..

[23] Cole S.P.C., Bhardwaj G., Gerlach J.H., Mackie J.E., Grant C.E., Almquist K.C., Stewart A.J., Kurz E.U., Duncan A.M.V., Deeley R.G. (1992). Overexpression of a Transporter Gene in a Multidrug-Resistant Human Lung Cancer Cell Line. Science.

[24] Kunická T., Souček P. (2014). Importance of ABCC1 for cancer therapy and prognosis. Drug Metab. Rev..

[25] Chan H.S., Lu Y., Grogan T.M., Haddad G., Hipfner D.R., Cole S.P., Deeley R.G., Ling V., Gallie B.L. (1997). Multidrug resistance protein (MRP) expression in retinoblastoma correlates with the rare failure of chemotherapy despite cyclosporine for reversal of P-glycoprotein. Cancer Res..

[26] Juszczyński P., Niewiarowski W., Krykowski E., Robak T., Warzocha K. (2002). Expression of the Multidrug Resistance-associated Protein (mrp ) Gene in Chronic Lymphocytic Leukemia. Leuk. Lymphoma.

[27] Wright S.R., Boag A.H., Valdimarsson G., Hipfner D.R., Campling B.G., Cole S.P., Deeley R.G. (1998). Immunohistochemical detection of multidrug resistance protein in human lung cancer and normal lung. Clin. Cancer Res. Off. J. Am. Assoc. Cancer Res..

[28] Zhang L., Song Y., Dai X., Xu W., Li M., Zhu Y. (2023). Inhibition of IDH3α enhanced the efficacy of chemoimmunotherapy by regulating acidic tumor microenvironments. Cancers.

[29] Zeng L., Morinibu A., Kobayashi M., Zhu Y., Wang X., Goto Y., Yeom C.J., Zhao T., Hirota K., Shinomiya K. (2015). Aberrant IDH3α expression promotes malignant tumor growth by inducing HIF-1-mediated metabolic reprogramming and angiogenesis. Oncogene.

[30] Cao G., Lam H., Jude J.A., Karmacharya N., Kan M., Jester W., Koziol-White C., Himes B.E., Chupp G.L., An S.S. (2022). Inhibition of ABCC1 Decreases cAMP Egress and Promotes Human Airway Smooth Muscle Cell Relaxation. Am. J. Respir. Cell Mol. Biol..

[31] Yeung B.H.Y., Huang J., An S.S., Solway J., Mitzner W., Tang W. (2020). Role of Isocitrate Dehydrogenase 2 on DNA Hydroxymethylation in Human Airway Smooth Muscle Cells. Am. J. Respir. Cell Mol. Biol..

[32] Zhao Q., Sun Z., Pan Y., Jing Q., Li W., Wang C. (2023). Role of ALOX5 in non-small cell lung cancer: A potential therapeutic target associated with immune cell infiltration. Zhong Nan Da Xue Xue Bao Yi Xue Ban = J. Cent. South Univ. Med. Sci..

[33] Kalayci O., Birben E., Sackesen C., Keskin O., Tahan F., Wechsler M.E., Civelek E., Soyer O.U., Adalioglu G., Tuncer A. (2006). ALOX5 promoter genotype, asthma severity and LTC4 production by eosinophils. Allergy.

[34] Mougey E., Lang J.E., Allayee H., Teague W.G., Dozor A.J., Wise R.A., Lima J.J. (2013). ALOX 5 Polymorphism associates with increased leukotriene production and reduced lung function and asthma control in children with poorly controlled asthma. Clin. Exp. Allergy.

[35] Hu Z., Bi G., Sui Q., Bian Y., Du Y., Liang J., Li M., Zhan C., Lin Z., Wang Q. (2020). Analyses of multi-omics differences between patients with high and low PD1/PDL1 expression in lung squamous cell carcinoma. Int. Immunopharmacol..

[36] Perretti M., D’acquisto F. (2009). Annexin A1 and glucocorticoids as effectors of the resolution of inflammation. Nat. Rev. Immunol..

[37] Yu C., Zhang L. (2022). Methylprednisolone up-regulates annexin A1 (ANXA1) to inhibit the inflammation, apoptosis and oxidative stress of cigarette smoke extract (CSE)-induced bronchial epithelial cells, a chronic obstructive pulmonary disease in vitro model, through the formyl peptide receptor 2 (FPR2) receptors and the adenosine 5′-monophosphate (AMP)-activated protein kinase (AMPK) pathway. Bioengineered.

[38] Jia W., Chen P., Cheng Y. (2019). PRDX4 and Its Roles in Various Cancers. Technol. Cancer Res. Treat..

[39] Nicolussi A., D’inzeo S., Capalbo C., Giannini G., Coppa A. (2017). The role of peroxiredoxins in cancer. Mol. Clin. Oncol..

[40] Park S.Y., Lee Y., Park J., Kim T., Hong S., Jung E., Ju Y., Jeong C., Park H.J., Ko G.H. (2020). PRDX4 overexpression is associated with poor prognosis in gastric cancer. Oncol. Lett..

[41] Ren R., Yuan Z., Xu Z. (2021). miRNA-144 targeting DNAJC3-AS1 reverses the resistance of the breast cancer cell line Michigan Cancer Foundation-7 to doxorubicin. Bioengineered.

[42] Starrett G.J., Luengas E.M., McCann J.L., Ebrahimi D., Temiz N.A., Love R.P., Feng Y., Adolph M.B., Chelico L., Law E.K. (2016). The DNA cytosine deaminase APOBEC3H haplotype I likely contributes to breast and lung cancer mutagenesis. Nat. Commun..

[43] Zheng J., Guo X., Nakamura Y., Zhou X., Yamaguchi R., Zhang J., Ishigaki Y., Uramoto H., Yamada S. (2020). Overexpression of PRDX4 Modulates Tumor Microenvironment and Promotes Urethane-Induced Lung Tumorigenesis. Oxidative Med. Cell. Longev..

[44] Dadachova E., Tong Y., Kikuhara S., Onodera T., Chen L., Myat A.B., Imamichi S., Sasaki Y., Murakami Y., Nozaki T. (2022). Radiosensitization to γ-Ray by Functional Inhibition of APOBEC3G. Int. J. Mol. Sci..

[45] Mizutani K., Guo X., Shioya A., Zhang J., Zheng J., Kurose N., Ishibashi H., Motono N., Uramoto H., Yamada S. (2019). The impact of PRDX4 and the EGFR mutation status on cellular proliferation in lung adenocarcinoma. Int. J. Med. Sci..

[46] Hao Y., Jiang H., Thapa P., Ding N., Alshahrani A., Fujii J., Toledano M.B., Wei Q. (2023). Critical Role of the Sulfiredoxin-Peroxiredoxin IV Axis in Urethane-Induced Non-Small Cell Lung Cancer. Antioxidants.

[47] Zhang T., Sang J., Hoang P.H., Zhao W., Rosenbaum J., Johnson K.E., Klimczak L.J., McElderry J., Klein A., Wirth C. (2025). APOBEC affects tumor evolution and age at onset of lung cancer in smokers. Nat. Commun..

[48] Ma C., Liu P., Cui S., Gao C., Tan X., Liu Z., Xu R. (2022). The Identification of APOBEC3G as a Potential Prognostic Biomarker in Acute Myeloid Leukemia and a Possible Drug Target for Crotonoside. Molecules.

[49] Ni T., Li Y., Guo D., Tan L., Xiao Z., Shi Y. (2023). LncRNA DNAJC3-AS1 promotes the biological functions of papillary thyroid carcinoma via regulating the microRNA-27a-3p/CCBE1 axis. Cell Biol. Int..

[50] Li H., Bi K., Feng S., Wang Y., Zhu C. (2022). MiR-140 Targets lncRNA DNAJC3-AS1 to Suppress Cell Proliferation in Acute Myeloid Leukemia. Mediterr. J. Hematol. Infect. Dis..

[51] Zhang Y., Li J., Luo B., Guo X., Liu J., Yang S. (2021). DNAJC3-AS1 Is Associated with Proliferation, Metastasis, and Poor Prognosis of Breast Cancer. Dis. Markers.

[52] Zhou M., Guo X., Wang M., Qin R. (2021). The patterns of antisense long non-coding RNAs regulating corresponding sense genes in human cancers. J. Cancer.

[53] Pereira M.A., Li Y., Gunning W.T., Kramer P.M., Al-Yaqoub F., Lubet R.A., Steele V.E., Szabo E., Tao L. (2002). Prevention of mouse lung tumors by budesonide and its modulation of biomarkers. Carcinogenesis.

[54] Veronesi G., Lazzeroni M., Szabo E., Brown P.H., DeCensi A., Guerrieri-Gonzaga A., Bellomi M., Radice D., Grimaldi M.C., Spaggiari L. (2015). Long-term effects of inhaled budesonide on screening-detected lung nodules. Ann. Oncol..

[55] Lim Y.X., Choo Y.N., Looi Y.T., Chuan Y.W., Chiam K.X., Wong R.S., Ng N.C., Goh B.H. (2026). Emerging Therapeutic Strategies in Asthma: Advances in Treatment, Drug Delivery, Drug Adherence, and Disease Management. Curr. Allergy Asthma Rep..

[56] Choi H.S., Kim S.-L., Kim J.-H., Lee D.-S. (2020). The FDA-approved anti-asthma medicine ciclesonide inhibits lung cancer stem cells through Hedgehog signaling-mediated SOX2 regulation. Int. J. Mol. Sci..

[57] Cannon M., Stevenson J., Stahl K., Basu R., Coffman A., Kiwala S., McMichael J.F., Kuzma K., Morrissey D., Cotto K. (2024). DGIdb 5.0: Rebuilding the drug–gene interaction database for precision medicine and drug discovery platforms. Nucleic Acids Res..

[58] Knox C., Wilson M., Klinger C.M., Franklin M., Oler E., Wilson A., Pon A., Cox J., Chin N.E., Strawbridge S.A. (2024). DrugBank 6.0: The DrugBank knowledgebase for 2024. Nucleic Acids Res..

[59] Oughtred R., Rust J., Chang C., Breitkreutz B., Stark C., Willems A., Boucher L., Leung G., Kolas N., Zhang F. (2021). The BioGRID database: A comprehensive biomedical resource of curated protein, genetic, and chemical interactions. Protein Sci..

