# Supplementary material for: Shared Transcriptomic Signatures and Network Interactions Between Lung Adenocarcinoma and Asthma
Source: Int J Mol Sci. 2026 Jun 19;27(12):5544. doi: 10.3390/ijms27125544 (PMC13299572; doi:10.3390/ijms27125544)
Supplement: Supplementary file 1 [file ijms-27-05544-s001.zip › Online Resource S4.pdf]

Shared transcriptomic signatures and network interactions between Lung adenocarcinoma and asthma

International Journal of Molecular Sciences

Seha AKDUMAN, Elif DÜZ, Merve GÜNDOĞDU, Didem TECİMEL, Altay Burak DALAN, Ömer Faruk BAYRAK, Didem SEVEN*

*Corresponding author affiliation: Department of Medical Genetics, School of Medicine, Yeditepe University, Istanbul, Turkiye

*Corresponding author email: didem.seven@yeditepe.edu.tr

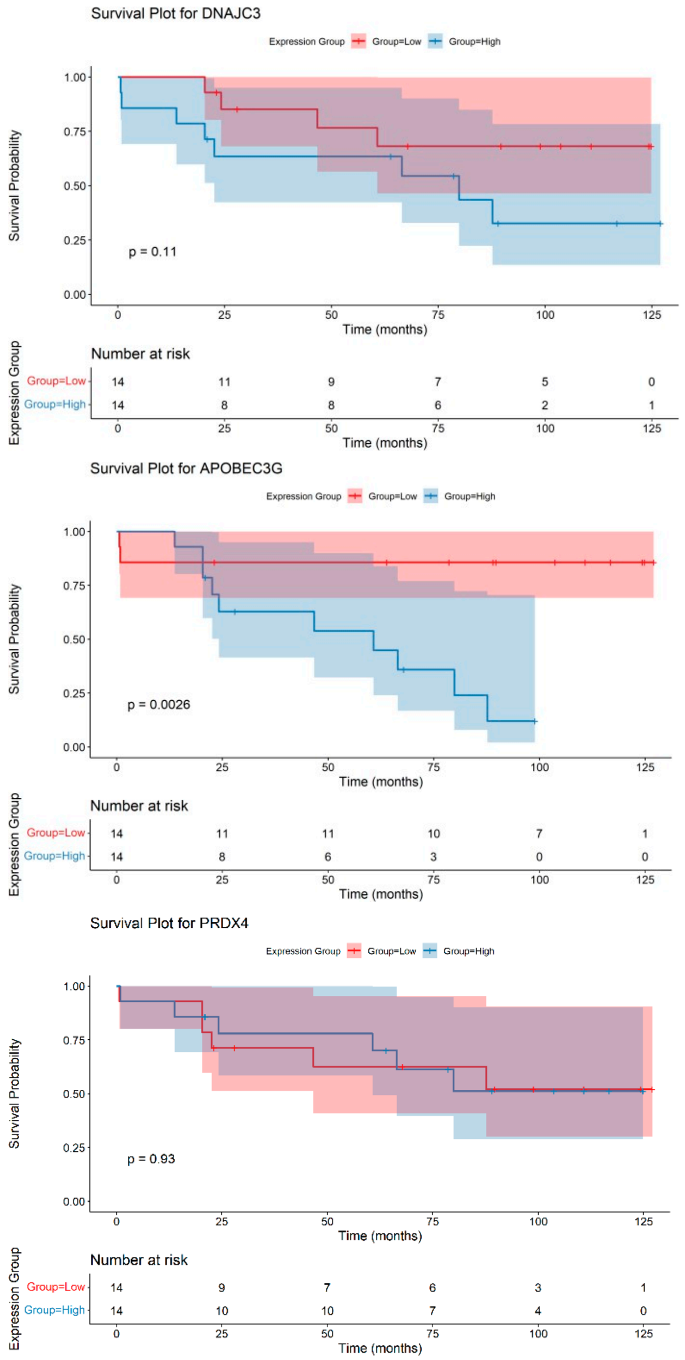

Supplementary Figure S1: The survival analysis plot for the selected genes in the GSE19188 dataset. This plot illustrates the relationship between DNAJC3, APOBEC3G and PRDX4 expression level and patient survival outcomes.

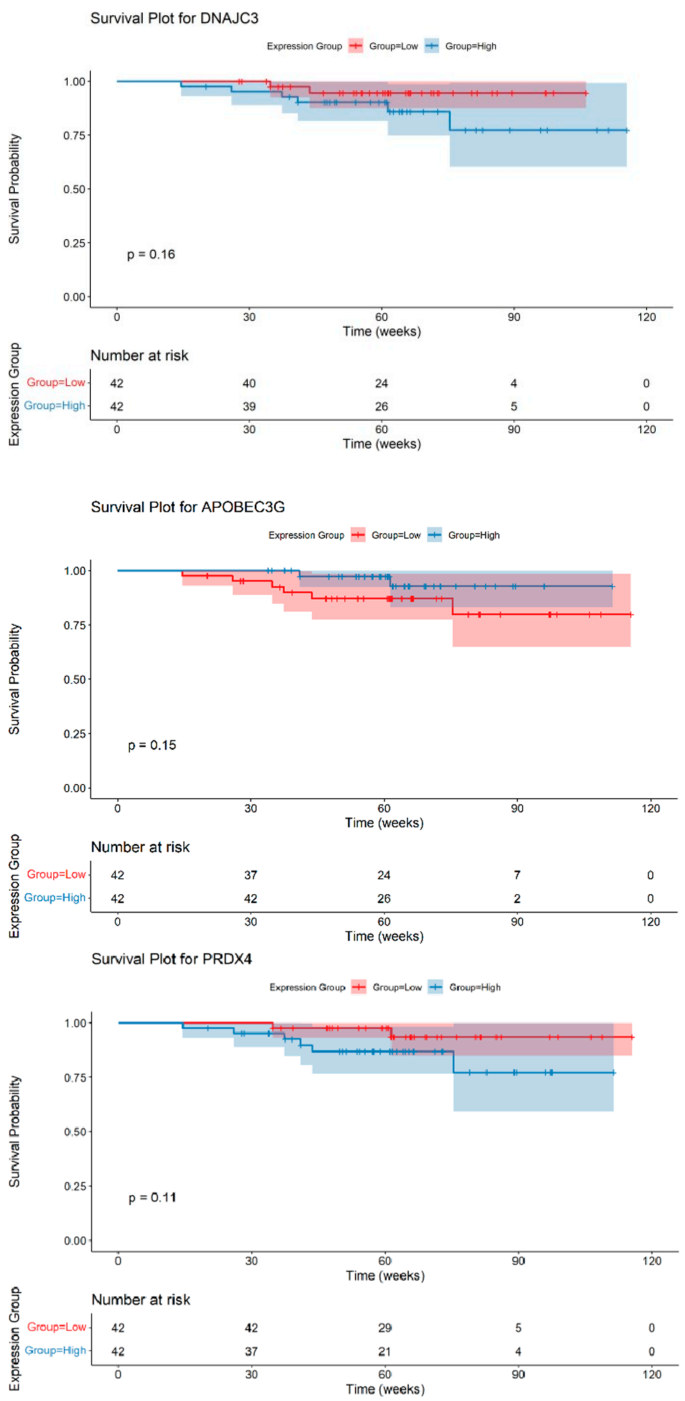

Supplementary Figure S2: The survival analysis plot for the selected genes in the GSE31210 dataset. This plot illustrates the relationship between DNAJC3, APOBEC3G and PRDX4 expression level and patient survival outcomes.
